# DIKEDOC: a multicriteria methodology to organise and communicate knowledge

**DOI:** 10.1007/s10479-022-04711-6

**Published:** 2022-05-21

**Authors:** Maria Franca Norese, Diana Rolando, Rocco Curto

**Affiliations:** 1 Department of Management and Production Engineering, Politecnico di Torino, Corso Duca degli Abruzzi 24, 10129 Turin, Italy; 2 Department of Architecture and Design, Politecnico di Torino, Viale Mattioli 39, 10125 Turin, Italy

**Keywords:** DIKEDOC methodology, Dispersed knowledge, Decision aiding, ELECTRE methods, Ivrea UNESCO site, Economic enhancement

## Abstract

DIKEDOC is a knowledge-based multicriteria methodology that is here proposed to organise dispersed knowledge about a complex problem when a decision process has not yet been activated, or is latent, and to generate an interaction space that produces new knowledge. An integrated use of logical and analytical tools is proposed, first for use at a technical level to organise any dispersed knowledge in a way that generates insights that can be communicated, and then in a participative context, to create an opportunity to interact, share personal points of view and experiences and to explore spaces of action, where such tools facilitate understanding, criticism and proposals. A pilot study was developed, by an interdisciplinary research team, in relation to the enhancement process of the “Ivrea, industrial city of the twentieth century” UNESCO site, which still needs to be activated after a long and complex decision process that led to the inclusion of the site in the World Heritage List. Several research activities and enhancement projects have been developed in the last few years, but a series of critical conditions have limited their implementation. A new perspective is now necessary to identify and control the uncertainties that have emerged, guide the incremental development of knowledge and foster relationships, decisions and policies. The paper presents DIKEDOC, a new knowledge organisation and problem description methodology, and the conducted pilot study, which led to the proposal of a constructive vision of decision aiding that logically and analytically “described” the space of action and its uncertainties.

## Introduction

Decision aiding is the activity of a person who, through the use of explicit, but not necessarily complete formalised models, helps another person or group of people to obtain elements of responses to questions posed by a stakeholder of a decision process (cf Definition 2.2, Roy, 1996).

The complexity and uncertainty elements of a decision problem and the nature and evolution of a decision process determine the character of a decision aid process and, therefore, the activities that have to be implemented and their sequence. Several factors can guide an analyst towards a specific methodological approach: the state of development and evolution of the decision process, the organisational complexity of the decision system and the operational complexity, above all the structuring level of the problem situation, the multiplicity and interdependency level of the problem issues and the resource state, with reference in particular to the time and to the availability and reliability of the data, information and knowledge (Norese, 2020a).

During a decision process, a great number of knowledge elements from disparate sources have to be organised and produced and then communicated through various modes of expression. Acquiring and organising these different kinds of knowledge could be critical decision aiding activities.

In recent years, knowledge has become a prominent theme in organisational literature (see, for instance, Kogut & Zander, 1996; Brown & Duguid, 2001; Carlile, 2004) and in strategic management literature (e.g., Nonaka & Takeuchi, 1995; Spender, 1996). Knowledge can be distinguished, in a decision aiding ambit, as formal knowledge about the use of tools and techniques, informal knowledge about social interactions, contingent knowledge about the nature, complexity and uncertainty of immediate situations, tacit knowledge about the process and history of the problem and experiences previously acquired, and meta-knowledge about the broader organisational, social and cultural setting (Keys, 2007). “The epistemology assumed in the literature tends to privilege the individual over the group, and the explicit over the tacit (as if, for example, explicit and tacit knowledge were two variations of one kind of knowledge, not separate, distinct forms of knowledge)” (Cook & Brown, 1999). The previous authors indicated that considering knowledge and knowing as mutually enabling, i.e. knowledge as a tool of knowing, can help explain how individuals and groups draw on all the different forms of knowledge and, more importantly, how the interplay between knowledge and knowing can generate new knowledge and new ways of knowing.

Modelling is the central activity of a decision aiding intervention, and the construction of models is a knowledge production activity (Déri et al., 1993). Acquiring and integrating different kinds of knowledge in a model are central decision aiding activities. However, such activities require significant cognitive efforts (Kolfschoten et al., 2014), not to mention the timing and costs associated with an investigation and knowledge organisation, which are not always consistent with the resources of a decision aiding intervention.

A model can be considered as the representation of a complex but well-defined reality or as a communication and reflection tool that allows the actors of a decision aid process to talk about a problem and to propose individual and/or organisational knowledge (Genard & Pirlot, 2002). The adoption of the former or latter interpretation may depend on the different decision aiding approaches: normative, descriptive, prescriptive or constructive (Tsoukiàs, 2007). The MultiCriteria Decision Aid (MCDA) methodology (see Roy & Vanderpooten, 1996 and the http://www.cs.put.poznan.pl/ewgmcda/ web site of the EURO Working Group MCDA) adopts a constructivist approach, where the as constructed model, the concepts, the procedures and their results become keys that are capable of opening certain locks for the decision process actors, and of allowing them to proceed in accordance with their objectives and systems of value (Roy, 1993). The concepts, models, procedures and results are developed, shared and criticised as a body of knowledge that evolves during the decision aid process. The analyst has the task of stimulating, orienting and monitoring the dynamics of this decision aid process.

Decision processes are often complicated, not only in terms of the participants, who may have different roles and attitudes, but also because interruptions and/or important changes in the decision system may characterise the process (Mintzberg et al., 1976; Nutt, 1993). Such a decision system includes decision makers and the actors involved in a decision process, as well as a decision structure that is formalised to a certain extent, with the actors' roles being recognised by the involved organisations and specific rules being implemented in the process. A limited formalisation level of the decision structure has a negative impact on the Decision Aid (DA) process, above all concerning uncertainty about the tasks and the action space.

Some DA interventions develop without having any direct relationship with a decision system, which the client of a DA intervention may know well because he/she habitually interacts with this system but has the aim of effectively proposing something completely new (see, for instance, Norese & Carbone, 2014). A DA intervention may also develop in relation to a decision system that is latent, because the decision process is in an initial pre-decisional state, or one that has not yet been activated because there is a difficulty that needs to be overcome in order to pass on to a decisional state of the process (Norese, 2020a). This may also occur because the decision process has been stopped, or it does not formally exist, while it has informally been activated in separate tables (Tsoukiàs, 2007). In these situations, the client may be someone who perceives the possibility of activating a new decision process or the need of reactivating or officially activating a process, or an actor in another related decision process, who perceives the need of a change in a decision system and in its overall vision of the problem.

Modelling through a constructivist approach consists in constructing a model *with* the person being aided to decide (Tsoukiàs, 2007), therefore different modelling and communication activities have to be implemented in relation to a latent or not yet activated decision system, to first create an interaction space between the analyst and knowledge sources and then with and between the possible actors in a new or re-activated decision process.

A methodology that is based on the agile and integrated use of logical and analytical tools is here proposed to operate in these situations, at a technical level, where knowledge is organised and validated, and at a participative level, where the technical results are communicated in order to be discussed and new knowledge is developed to generate a shared description of the decision problem and some hypotheses of action. Such a methodology may be used in different contexts, but it is above all oriented towards situations that belong to a new field that is called “policy analytics” (see Tsoukiàs et al., 2013; Abi-Zeid & Tremblay, 2016; Daniell et al., 2016; De Marchi et al., 2016). However, the aim of such a methodology is that of being a driving force in a process that is not one of designing or evaluating policies but rather of allowing and facilitating the definition of a space of action and a decision system that wants to or has to design a policy.

Some MCDA interventions were characterised in the past by a request for new modelling and communication activities in relation to a latent or not yet activated decision system (see, for instance, Norese, 2009; Norese & Carbone, 2014; Norese et al., 2015a, 2015b), or innovative uses of multicriteria methods were requested by experts in specific domains who had acquired a great deal of data in relation to a project and needed to generate knowledge and transform data into information (see Balestra et al., 2001; Cavallo & Norese, 2001; Norese, 2020b). These past experiences created the basis for a general approach and facilitated the study and action, and then the proposal of a new methodology, when a request arrived from one of the technical actors in the long and complex decision process that resulted in the inclusion of the “Ivrea, industrial city of the twentieth century” site in the UNESCO World Heritage List in 2018. This important result, which was considered an opportunity for the complex socio-economic context of the city and its surrounding area, could now be lost because of a series of critical conditions, including some economic and political constraints which have been exacerbated by the COVID-19 emergency. The process needs to be reactivated to enhance this site of great historical, architectural and environmental value, but a decision system is latent.

Several useful knowledge elements were acquired in the years between 2014 and 2018: data on the built environment and on the socio-economic and cultural context and territory, documents pertaining to the candidacy process, the points of view of the involved actors and the needs and goals of the organisations that could now play a possible role in the site enhancement process.

A pilot study was activated by an interdisciplinary research team, in response to a request for a methodological aid in the activation of the site enhancement process, to organise the dispersed knowledge and to use the results to communicate with the potential actors of the new process.

The first section of the paper describes the proposed knowledge organisation and communication methodology, in relation to literature dealing with the subject. The second section presents the problem that stimulated the pilot study and this work. Some knowledge organisation results that had been achieved at the end of a technical application of the methodology are included in the third section, as they were presented to the Municipality of Ivrea. An example of how multicriteria models can integrate, visualise and communicate organised knowledge, and how multicriteria methods can indicate possible uses of this knowledge, is presented in the fourth section. Some considerations on the nature of this approach and on the results of the pilot study are proposed in the fifth section and in the conclusions.

## The methodology

The methodology proposes an approach that logically and analytically deals with contingent knowledge about the nature, complexity and uncertainty of a complex problem situation, to “describe” the space of action and its constraints and uncertainties and to propose this description in a participative context, where tacit knowledge in relation to the history of the problem and experiences previously acquired, and informal knowledge about social interactions can be expressed and included in a description, to change and improve it until a legitimate description is generated.

Disparate sources of knowledge can be present in relation to a complicated problem (in literature on the field or other connected fields, in the involved organisations and the territory, in the practice of some of the stakeholders and so on), and can be expressed through different modes (documents, norms, newspaper articles, analyses and reports, storytelling; interviews, diagrams, ideas, images, networks; structured or unstructured data about design concepts, assertions, proposals, suspects, protests, judgements and so on) and through different languages (social, political, juridical, scientific, technical, economic, administrative and so on). Only an integrated and agile use of different tools can generate an organisation of different kinds of knowledge, languages and expression modes, and its structured inclusion in models that can be easily communicated, discussed, validated or improved, used to foster relationships between people and organisations, and to generate new knowledge, models, discussions and elaboration of stimuli for an enhancement action.

Methodologies and tools have been proposed in literature to help analysts and decision makers to understand the visions and perceptions, purposes and motivations, values, resources and relationships of a complicated problem context. Hermans and Thissen (2009) proposed several actor analysis methods in relation to the different ways of focusing on the specific characteristics of the actors. Analyses have been developed to understand why the current practices work effectively in facilitating shared understanding, mutual learning and rigorous analysis of knowledge frameworks, and how they can be further improved (see for instance Kolfschoten et al., 2014; Ormerod, 2014). Visualisation techniques, ranging from modelling languages (such as SADT, Structured Analysis and Design Technique, or UML, Unified Modelling Language) to different kinds of diagrams, can be used to organise and describe knowledge.

Acquiring and integrating different kinds of knowledge are decision aiding activities. Visualisation tools and Problem Structuring Methods (PSMs) are used for these purposes in the field that is called soft Operations Research (soft OR), while analytical models and applications of methods are developed to integrate knowledge in the hard or classic OR field.

Kotiadis and Mingers (2006) described the paradigms that are the basis of the two fields and analysed the barriers and potentialities of a multimethodology that includes both soft and hard approaches. Combining methodologies of a different nature may facilitate decision aiding, but also implies that the stakeholders must understand and acquire different languages and ways of thinking. A multimethodology could generate difficulties and misunderstandings in the decision context, for example when some components of a PSM are used and integrated with another PSM and could require specific attention to cognitive and cultural obstacles (Mingers & Brocklesby, 1997; Rosenhead & Mingers, 2001).

PSMs are being used more and more frequently to satisfy the cognitive aim of clarifying and formulating a decision problem in complex situations (see, for instance, Rosenhead, 1989; Mingers & Rosenhead, 2004; White, 2009). Multicriteria decision analysis methods are often combined with PSMs (see Belton & Stewart, 2010 and the review by Marttunen et al., 2017). The sequence of a multicriteria application, after the structuring contribution of a PSM, draws attention to the complementarity of these approaches (see, for instance, Belton et al., 1997; Bana e Costa et al., 1999; Montibeller et al., 2008; Norese et al., 2004, 2008; Stewart et al., 2010; Ferreira et al., 2011).

The methodology that is here proposed underlines the need for an integrated (and not sequential) use of logical and analytical tools, not to obtain elements of responses to questions posed by a decision maker in a decision process but to organise the available or acquired dispersed knowledge in relation to a problem situation and to put the *description problematic* (P.δ) into operation. P.δ “poses rather than solves a problem”, defines and formulates the problem “properly in terms that are acceptable to the various actors” and “allows the effort to be constructed rigorously and systematically, yet in a fashion that is straightforward and understandable by most of the actors” (Roy, 1996). Multicriteria models allow knowledge to be translated into intelligible syntheses that analytically deal with different aspects in a transparent and formal language (Genard & Pirlot, 2002). These models are used to make a shared vision explicit or to analytically express multiple points of view and preferences produced by the development of a decision process (Ostanello, 1997), although they are above all used in this methodology as models of concepts, which do not have the aim of representing an external reality but only how things may be seen from a technical point of view, and how a shared or multiple vision may be expressed and discussed in a participated context (Mingers & Brocklesby, 1997).

Models and applications of methods are used in the proposed methodology to identify what elements of knowledge are not present in the problem description or those that have to be redefined. They describe how a decision maker can deal with a problem and what elements of a preference system should be elicited and used to analyse the consequences of a decision implementation (Norese, 2009) and how working hypotheses (Ostanello, 1997; Roy, 2010) can be described, starting from the available or collected empirical knowledge, and without any preference expression of the decision maker(s) (Bouyssou et al., 2006).

Multicriteria (MC) methods are used in decision aiding to compare the actions that are evaluated in MC models, in relation to a specific request or problem statement (to choose the best compromise, rank the actions or assign them to categories) (Roy, 1996). Different MC methods have been proposed in literature (see, for instance, Figueira et al., 2004; Ehrgott et al., 2010) and should be chosen in relation to the nature of the decision problem and the expected results, and to a set of specific requirements in relation to the decision context and its sources of information and knowledge (Roy & Słowinski, 2013). The proposed methodology first resorts to outranking methods that allow multiple languages, each one associated with a criterion that expresses a technical, social, political, juridical or administrative aspect, to be used directly and visualised.

The formal, explicit and transparent language of multicriteria models and methods, in interaction with logical and visualisation tools, is here proposed to organise dispersed knowledge, when the methodology is activated at a technical level, and to describe the resulting knowledge at a participative level, to produce new knowledge in a sequence of learning cycles and to generate a shared description of the decision problem and possible actions of a decision system.

DIKEDOC (DIspersed KnowledgE: Describe, Organise, Communicate) is the acronym of a methodology that is associated with a constructivism path and is based on the communicative vision of rationality that contributes to the reflexive decision aid approach (Meinard & Tsoukias, 2018). DIKEDOC focuses on elements of knowledge (concepts, models, procedures and results) that become “tools for developing convictions and allowing them to evolve, as well as for communicating with reference to the bases of these convictions” (Roy, 1993).

### DIKEDOC (DIspersed KnowledgE: describe, organise, communicate)

A DIKEDOC application includes two different phases, both of a collaborative nature, the first of which is developed at a technical level and the second at a participative level. Communication and recursive activation activities of previous steps are present in both phases.

When DIKEDOC is activated at a technical level, three main steps and several activities are developed. Logical and visualisation tools are used to acquire and synthesise a confused set of knowledge, from different sources and in relation to the problem situation. In the second step, the same tools allow the analysed knowledge to be organised, visualised and tested. The two steps produce new knowledge, whose quality is discussed at a technical level until it can be summarised in a manageable way and reused in a different context (Majchrzak et al., 2004).

The activities of the third step allow the organised knowledge to be transferred to analytical models, through a continuous passage from organised and visualised knowledge to local formalisation of some aspects and their integration in analytical models. MC models and methods are used in this step to verify the consistency of the knowledge included in the models, and to validate the quality, completeness and usability of the available knowledge or to orient new activities of knowledge acquisition and/or organisation.

The technical phase of DIKEDOC produces different kinds of results: problem formulation, complexity simplification, uncertainty elimination (or at least understanding and control), knowledge testing, as achieved through its use in relation to a model and specifically to the definition of actions and criteria, and applications of MC methods, in terms of analytical procedures and their results to propose for discussion in technical and non-technical contexts. All the different results, when validated at a technical level, are communicated and proposed for discussion in the participative phase.

The activities of the first step of the new phase consist in collectively analysing the elements of the problem formulation, by means of logical and visualisation models, and validating or improving them at a participative level, with the creation of an interaction space. Participants can reflect on single elements and on their overall consistency, quality and completeness.

The second step activities mainly consist in analysing the MC model(s) and MC method application(s) and results, which have been constructed at a technical level without any expression of preference parameters (or with some declared parameters that are only connected to technical needs of uncertainty control and balance between the main aspects of the model). These activities have multiple aims: the analysis of the multiplicity of points of view, made explicit during the analysis of the problem formulation, and of any expressed need of uncertainty control, a critical analysis of models and results, the proposal of a new MC modelling (possibly in relation to a new problem formulation) with explicit expressions of visions and preferences, the understanding of the potential use of the organised knowledge by means of models and methods, generation and definition of a specific action space and identification of some hypotheses of action.

MC models and methods are used in DIKEDOC as schemas that have been built in a *simulative way* (Norese, 2020b), in a learning context of understanding construction (Brown & Duguid, 1991), to propose and test some working hypotheses, which Roy (1993) described using the metaphor of “a set of keys which will open doors for the actors”. Models, and the application of methods and results, can easily be submitted to critical discussion because they lead to dialogue, conditions of mutual understanding and mutual learning (Genard & Pirlot, 2002).

When this path produces results, specific questions in relation to the validity of these results require a formal, transparent and “quick” answer, at both a technical and a participative level. When, for example, a result consists of an identified decision scheme, which could become a consistent and effective strategy, and some projects can be activated in relation to this strategy, their evaluation and comparison could confirm the validity of the strategy or suggest its improvement. MC methods and their SW tools are applied to facilitate “quick” answers.

The integration of different tools is facilitated by the proposal of a unifying language that proposes a criterion and its modelling to any knowledge source or stakeholder as an example of how a perception, concern or doubt can be formally dealt with, when these elements could be neglected because not numerically significant.

Such a unifying language indicates that the conflictual points of view can co-exist in a model, while contradictions have to be identified, analysed and resolved.

The logical and analytical tools used for such an integration are consistent since they share the same basic assumptions, ways of thinking and knowledge vision, and their procedural logic is flexible and compatible with the aim of developing, testing and evolving a knowledge base that facilitates the elimination or control of the most preeminent uncertainties, the comprehension of a clarified situation and some proposals of activities and new relationships. The procedural rationality of MC methods facilitates a description of how organised knowledge can be used and how a method can produce valid results, but also of how specific knowledge conditions or actors’ behaviour may imply the exclusion of a method or orientate the user towards a different procedure or method.

The organisation and representation steps first allow the analysts and knowledge sources, and then the stakeholders to explore the nature of the problem, to distinguish and recognise its main elements and to be involved in a process of interpretation and communication. The results of a continuous shift from one kind of tool to another, in both the DIKEDOC phases, are an incremental knowledge organisation and problem formulation.

A complex problem of site enhancement is presented in the next section, together with an introduction to the pilot study in relation to the problem.

## The Ivrea UNESCO site enhancement problem

Adriano Olivetti was the founder of an innovative ‘community’ company model (Olivetti, 1945). He not only created new products (such as the Lettera 22 portable typewriter, which is on show at the MoMA in New York, and the P101, the first personal computer in the world), but also had a wealth of ideas about work and culture and proposed the concept of a factory as a place that should put people, their aspirations, skills and values at its centre. Olivetti’s factories were designed with in-built spaces for cafeterias, playgrounds, rooms for debate and film screenings, and libraries with tens of thousands of books and magazines. In addition to the Olivetti factory, a series of buildings were built for the workers and managers, and an extended network of social services was created, including nursery schools, the first hospital in Ivrea and a leisure centre.

**Fig. 1:**
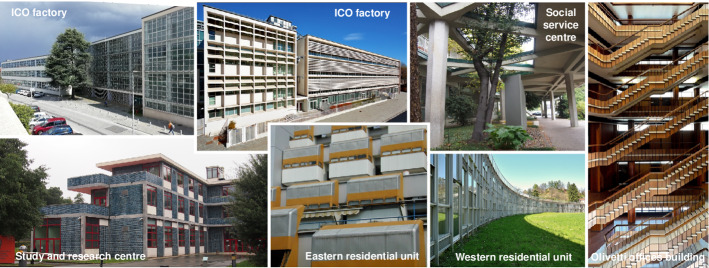
Some of the “Ivrea, an industrial city of the twentieth century” UNESCO site buildings (Source: elaborated by the authors)

The “Ivrea, industrial city of the twentieth century” UNESCO site is a large unitary urban system of high historical, architectural and environmental value, consisting of more than 100 well-preserved buildings that represent manifold expressions of Modern Heritage and different building types (some examples of which are shown in Fig. 1). The Olivetti built heritage is outstanding (see http://www.fondazioneadrianolivetti.it/index.php), since Olivetti commissioned out the works to famous architects, who were representative of the Italian “Modern Movement”, and who designed different building typologies that were closely integrated with their surrounding outdoor spaces, green areas and infrastructures.

Currently, 98% of the assets belong to private subjects, while just the kindergarten and a space in the factory are public properties that belong to the Ivrea Municipality. The owners of the assets obviously want to obtain the maximum profit from their properties and the Municipality of Ivrea is playing a mediating role between them and the Superintendence, who controls the application of the imposed constraints. It has been decided that the several residential buildings have to be restored without any changes to their original use. Most of the other spaces in the UNESCO site are unused or underused, and therefore require redevelopment through the transformation of their original functions. However, any enhancement action will need to include restoration and will involve economic-financial issues. Such an action will have to be both compatible with the historical and architectonic values of the assets and include the establishment of new functions, identified in relation to the potential demand segments and based on their economic and financial feasibility.

The document with which the UNESCO World Heritage Committee (WHC) motivated the inclusion of the site in the UNESCO World Heritage List (UNESCO WHC, 2018) underlined the value of this heritage as testimony of an era and of a modern vision, which is emblematic of the transition from mechanical to digital production. Nevertheless, the WHC also highlighted the uncertain future of many buildings, due to their maintenance level and state of deterioration, and that efforts would be necessary to develop new uses that were at the same time similar to the original ones (such as telecommunications, production or cultural activities). The WHC requested “a strategic conservation plan for the property, including the planned conservation outcomes for each building, strategies for new uses of vacant buildings, and resources for maintenance”.

Any decision in relation to the enhancement of the site will be difficult. This situation became evident during the five years of analysis at the Politecnico di Torino in laboratory activities on Heritage preservation and enhancement, a didactic and research experience (Curto et al., 2022a) activated together the Municipality of Ivrea (represented by the Mayor, City planning Councillor, Coordinator of the UNESCO nomination, Coordinator in charge of Demographic Services, Communication and Information Systems) and the Superintendence of Archaeology, Fine Arts and Landscape for the Metropolitan City of Turin (represented by officials who were also involved as professors of the “Heritage preservation” module). Furthermore, the students that took part in the activities interacted with the local association of industries (Confindustria Canavese), as well as with some companies in order to acquire specific data that would be useful to correctly set the economic-financial analyses and verify the feasibility and profitability of their projects. A great deal of knowledge was acquired about the site, its value and its enhancement complexity. Some data, which were acquired and structured in a GIS, were used during the candidacy process, while all the other unstructured and inhomogeneous pieces of knowledge ran the risk of vanishing. However, the organisers of the laboratory activities thought that the knowledge should be systematised and made accessible to the actors involved in the enhancement process, and above all to the Municipality.

### A pilot study to organise and communicate dispersed knowledge

A DIKEDOC application was developed at the Politecnico di Torino, in an interdisciplinary research context, at the end of five years of laboratory activities and analyses on the UNESCO site. Researchers from different disciplines were involved (economic evaluation, multicriteria decision aiding, public policy analysis, architectural restoration) for two main reasons. The relationships between the concepts of preservation, conservation, reuse and economic-financial sustainability are complicated and therefore require a multidisciplinary approach. Moreover, the rich documentation acquired and produced during the laboratory activities could only be proposed to stakeholders in the area if organised and validated from the different technical points of view, which needed to be made explicit.

The DIKEDOC application involved the interdisciplinary team that used the dispersed knowledge which had been acquired and created in the laboratory to build and share a description of the situation and its complexity. Knowledge organisation, visualisation and discussion steps were integrated with other steps in which any topic of discussion in the interdisciplinary team became a formal model to test the included knowledge, reduce possible misunderstandings and describe how possible actions and decisions could be evaluated and compared by means of analytical methods.

Some logical tools were used in the first steps to clarify the nature of specific difficulties, to identify the main uncertainties that were making any decision complex or impossible and to use the acquired knowledge in order to generate possible answers to some central questions. The internal logic and understandability of the results was tested by individuals who had not been involved in the laboratory activities.

Different MC models were then developed in relation to the central questions and associated problems, and MC methods were applied to the models to test the quality and usability of the organised knowledge, and to generate examples that could be explained and discussed in a participative context.

Some of the steps and results of the DIKEDOC application conducted at the technical level were presented to the Municipality, the key actor in the site enhancement, to verify the reliability of the organised knowledge and discuss and test its significance in consideration of the involvement, in a participative context, of some potential actors’ in a process of site enhancement. The presentation only included the essential steps and results, in order to facilitate an easy transmission of the logic and potentiality of the methodology. Some knowledge organisation results, as they were presented to the Municipality of Ivrea, are described in the next section.

## A DIKEDOC application at a technical level

A PSM, the Strategic Choice Approach (SCA), was used in the first iterations of the DIKEDOC application, which only involved the interdisciplinary team, in order to share the dispersed knowledge of the main problems and uncertainties that were making any decision complex (some of the procedural iterations are synthetically described in Curto et al., 2022b). SCA was proposed (Friend, 1989; Friend & Hickling, 1987) as a method for complex and unstructured decision problems, and it is normally used to structure problems characterised by a high level of uncertainty with several interconnected decisions, to explicitly manage and reduce complexity, control uncertainties, exclude alternatives that are not feasible for technical or political reasons, and elaborate and compare compatible solutions. The SCA methodology, with the support of its STRAD software tool, has been applied in various situations (see, for instance, Montibeller & Franco, 2011; Han & Laiô, 2011; Norese et al., 2015a, 2015b; Rolando, 2015; Georgiou et al., 2019; Paucar-Caceres et al., 2020).

After some iterative applications of SCA, to organise the acquired knowledge and reduce complexity and uncertainty, the situation was considered complex and rich in interconnected aspects and decision needs, and therefore other simpler tools were used to synthesize and describe the results and verify the internal consistency of this technical description of the problem situation. The results were the identification of the central questions, interconnected with all the other questions, and of the issues that could be dealt with, at a technical level, by means of some components of SCA that could easily produce communicable results.

The knowledge organised at the technical level and “made communicable” is here proposed, together with the tools that were used, and some SCA results are presented in Sects. 3.2, 3.3 and 3.4. These results were then used as inputs for MC models and method applications, details of which are presented in the fourth section.

### Structure of the stakeholders and the main problem issues

**Fig. 2:**
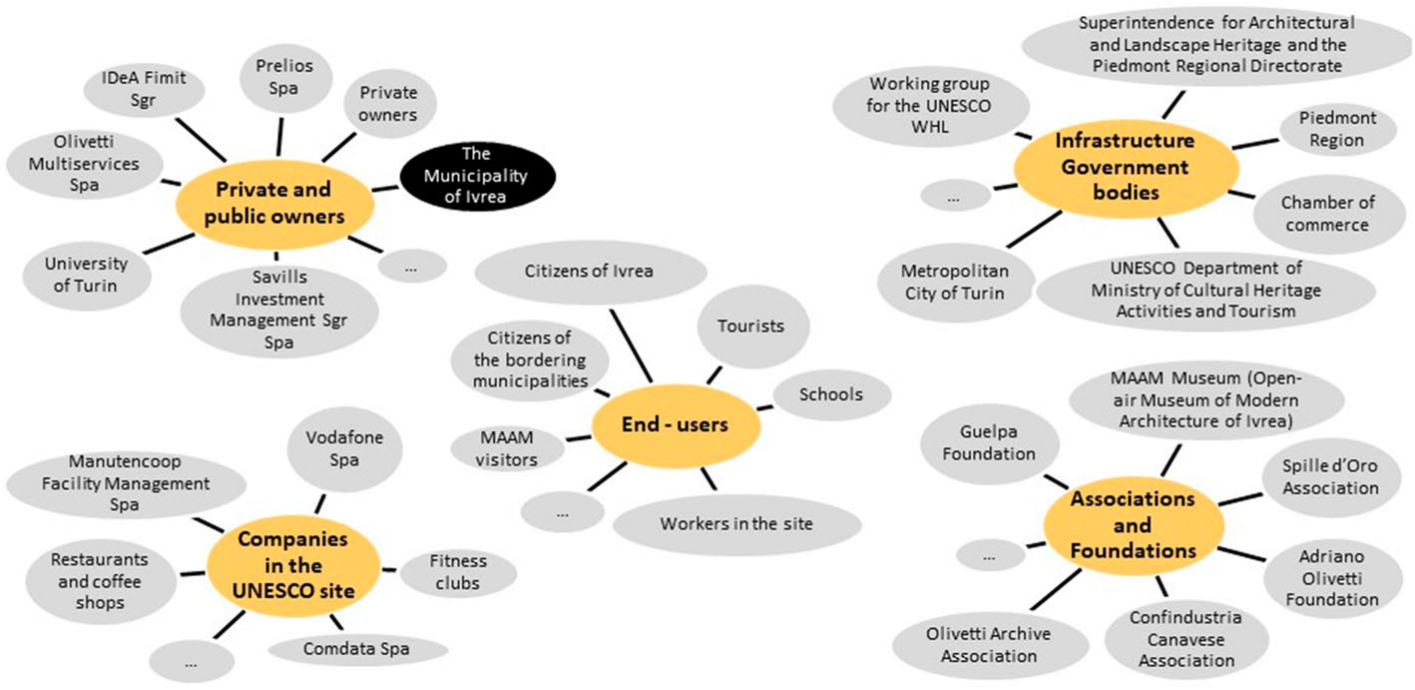
Structure of the stakeholders (Source: elaborated by the authors)

A large number of public and private stakeholders could or should be involved, at different levels and with different relationships and possible roles, in the site enhancement and management process. These figures are continuously evolving, and a distinction into five typologies facilitated a general overview of the stakeholders’ structure (Fig. 2), where both the private and public owners of the assets that have to be redeveloped and enhanced play important financial roles.

Not all the stakeholders are interested in the overall enhancement problem, but their points of view, in relation to some important aspects, had to be considered. Some their needs, goals and strategies were identified. Particular attention was paid to the role of the Municipality of Ivrea, which should coordinate the different stakeholders and control the enhancement and redevelopment process of the whole site.

The Politecnico di Torino, as one of the technical actors in the decision process that led to the inclusion of the site in the World Heritage List (WHL), identified a series of complexities and uncertainties that could have made any new decision on the site enhancement difficult and risky. These difficulties were above all related to the restoration issues and financial problem of funding the site enhancement, but also to how the requests and recommendations of the UNESCO World Heritage Committee (UNESCO WHC, 2018) could be accomplished and how the Outstanding Universal Values fixed at the time of inscription in the WHL could be conveyed (ICOMOS, 2011). The complexity and uncertainties associated with enhancement of the site generated specific research questions which were dealt with in Master theses and during a Ph.D. course; these detailed analyses produced a new perception of the situation.

**Fig. 3:**
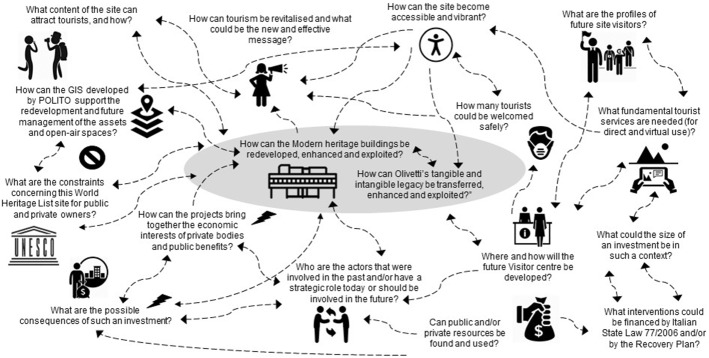
A “rich picture” diagram of the main questions (Source: elaborated by the authors)

Several questions that had arisen from the laboratory activities and detailed analyses were dealt with and discussed in the first phase of the DIKEDOC application and then synthesised in a “rich picture” diagram, which included the needs, expectations and concerns of the main actors (Checkland, 2001) and was used to interconnect questions (Fig. 3).

Some of these questions are related to the nature of tourism and its requirements and they have direct consequences on the kind of services that the future visitor centre and the city of Ivrea should provide to attract tourists and people in general and thus revitalize the urban area. The COVID-19 pandemic has had a heavy impact on the touristic role of this site, which had already been considered a great challenge because of the economic fragility of this area before this health crisis erupted. Other doubts are related to the lack of availability of public and/or private funds, which should be associated with strategic interventions to generate both profitability for the private investors and public benefits. Moreover, some questions are related to norms, information and activities that should be compatible with the historic and cultural value of the assets, in agreement with the owners.

**Fig. 4:**
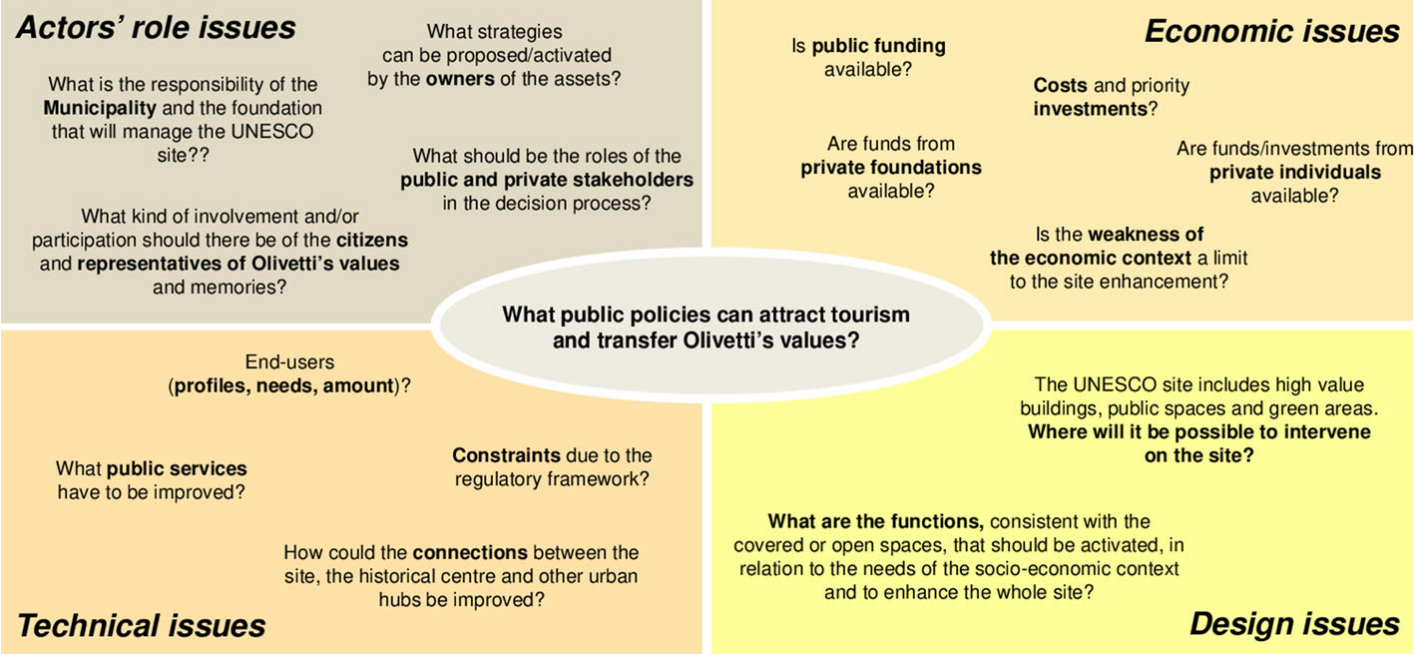
The evolution of the “rich picture” diagram: from questions to issues (Source: elaborated by the authors)

The diagram in Fig. 3 was analysed and the two central questions, which should always be considered when dealing with any other question, were synthesised as “What public policies could attract tourism and transfer Olivetti’s values?”. The other questions were associated with four kinds of issue: the actors’ roles, and economic, technical and design issues (Fig. 4).

Some issues outlined in the figure are associated with the possible roles of some actors (the Municipality of Ivrea, the owners of the assets, citizens and representatives of Olivetti’s legacy) in a transparent and inclusive decision process, which will be able to point out and solve both political and technical issues, the latter being related, for example, to building constraints, as well as to connections and public services that will need to be implemented in and around the site. The stakeholders’ structure in Fig. 2 underlines how complex the context is.

The economic issues that limit the enhancement process of the site were highlighted, with particular reference to the critical condition of the economic context, which is characterised by limited public resources, and balanced by the presence of private individuals who are willing to invest in the redevelopment of some buildings, but probably concerned about the overall amount of the necessary investments and the current state of the real-estate market, which is very weak at present (Barreca et al., 2022).

Issues linked to technical aspects were studied during the laboratory activities, in relation to the elaborated projects. Soma data on the physical characteristics of the built environment, as well as data concerning the territorial, socio-economic and cultural context, had been easily acquired and structured in a GIS (Barreca et al., 2017). The GIS was continuously updated, and it can now be used as a component of a knowledge base (Barreca et al., 2020), which should include studies on the restoration of buildings for specific reuse, in relation to public policies and actions for the enhancement of the whole UNESCO site.

The Design issues were chosen to be analysed in depth in the pilot study, to consider some possible activities and policies that the Municipality of Ivrea could implement, starting from the questions: WHERE is it possible to intervene on the site? and What are the FUNCTIONS, consistent with the covered or open spaces, that should be activated, in relation to the needs of the socio-economic context and to enhance the whole site?

Some components of SCA, above all the Designing and Comparing operation modes, were only used when they were essential to reduce complexity and uncertainty and to produce easily communicable results.

### Generation of alternative options and their compatibility analysis

The Analysis of Interconnected Decision Areas technique, which is a Strategic Choice Approach (SCA) tool, was used to analyse the two Design issues that were dealt with in the WHERE and FUNCTIONS decision areas. A set of options was defined for each decision area and their compatibility was explored.

Three classes of buildings, with similar characteristics in relation to the space distribution and constraints about some specific reuses, were recognised as possible options for the first decision area. A fourth class, pertaining to all the green areas and open-air spaces that could be used to enhance the urban system and to generate positive impacts for both private and public stakeholders, was identified.

**Fig. 5:**
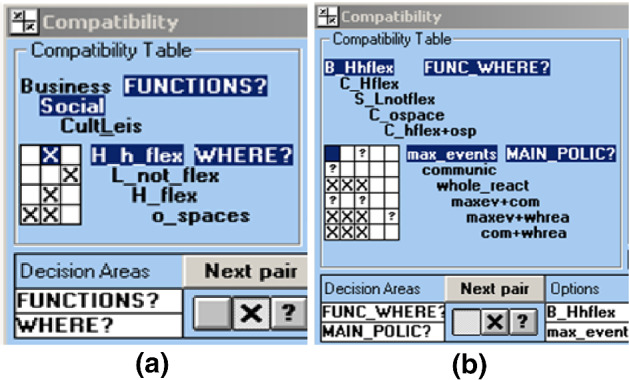
Compatibility analyses (where X means incompatibility; a blank square means compatibility; ? means uncertainty that requires investigation or an expert’s judgement) (Source: elaborated by the authors by means of the STRAD SW tool)

Three of the second decision area options were associated with three kinds of function (Business, Social and Culture, and Leisure) and the “validity” of each combination of function and class of assets was analysed, in terms of risks and potentialities, in the Compatibility analysis of the SCA Designing mode (see the Compatible table of the STRAD SW tool in Fig. 5a).

Seven combinations of functions and asset classes were considered compatible, and their consistency was analysed in relation to the present situation in the site, where the business function has already been implemented in the buildings with huge, highly flexible spaces (Hhflex), and to the possibility of implementing the cultural function in a combination of huge flexible spaces (Hflex) and open spaces.

A new decision area was then analysed, and a set of options was associated with the main issue: “What public policies could attract tourism and transfer Olivetti’s values?”. Five compatible combinations of assets and functions were analysed in relation to three policy options that the Municipality could implement, together with three combinations of these options (Fig. 5b).

The Compatibility analysis in Fig. 5b produced some useful indications about the compatibility of cultural and leisure activities and the proposed policies, but also underlined the difficulty of an analysis of the possible policies in a technical context. The compatibility between some policies and functions was sometimes uncertain and this result suggested the activation of another SCA tool, the Comparing mode, that was used to further analyse the identified possible policies, as described in Sect. 3.3.

### Analysis of the possible policies

Three possible public policies were identified by the technical team to improve the attractiveness of the UNESCO site and to increase the number of visitors. One of these policies is *Maximising the enjoyment of the site through events*, which could be organised in open spaces or huge buildings in a sustainable way, while taking into account the necessary post-COVID19 measures. Another is *Mainly working on the communication of Olivetti’s legacy values*, which are not only architectonical and/or historical values, but also a combination of the cultural and political concepts of “a human-based industrial site” and “innovation sustainability”. Another policy concentrates on the *Re-activation of the whole site, which implies project integration in a systemic vision of the UNESCO site*. More complicated policies may include two of these individual visions and policy aims.

The Comparing mode of SCA has the aim of stimulating decision makers to identify certain aspects and use them to distinguish and select the alternatives, or at least eliminate the least interesting. Such aspects are used as conceptual tools that facilitate the expressions of points of view, in terms of comparative judgements. Therefore, these aspects have been defined as Comparison Areas (CAs).

The identification of CAs, in DIKEDOC and specifically in this case, is an activity that is solicited as a result of some difficulties that a compatibility analysis may have encountered and underlined. The use of CAs to express comparative judgements from a technical point of view facilitates the definition of “abstract” elements that are not clearly delineated at a technical level without any explicit political indication.

**Fig. 6:**
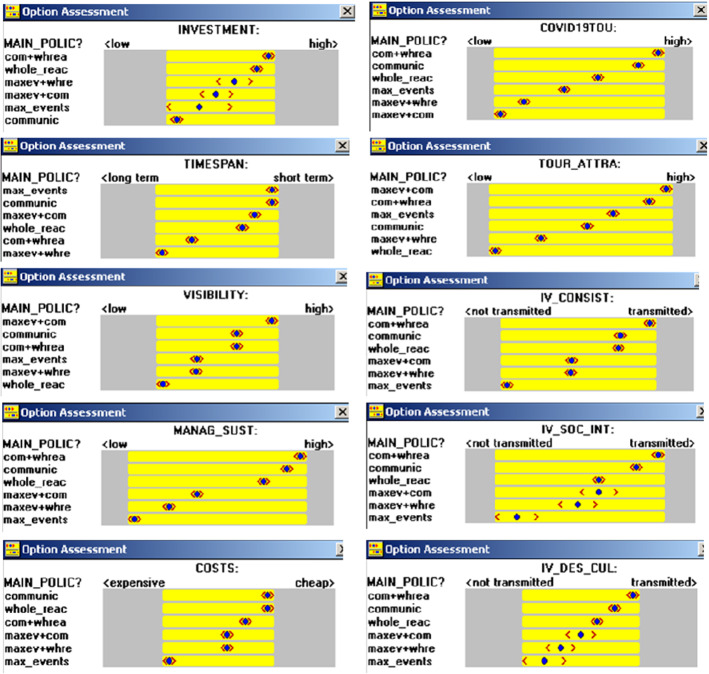
Option Assessment windows (Source: elaborated by the authors by means of the STRAD SW tool)

In this case, the potentialities and limitations of any individual policy or combination of possible policies were analysed to establish the potential effectiveness and associated risks, above all in relation to the current post-COVID19 measures, and the consistency of such policies with Olivetti’s vision. The analysis of the latter aspect was not easy, but it generated a better definition of the policies and, in general, of the possible enhancement actions. The CAs identified and analysed by the interdisciplinary team are indicated in Table 1. The Option Assessment windows of the STRAD SW tool allowed us to visualise whether the comparative judgements could distinguish the compared options, i.e. individual and combined policies (see Fig. 6).

**Table 1:** Comparison areas

INVESTMENTS	Capacity to attract public and private investments
TIMESPAN	Time necessary to achieve some of the first expected results after a policy activation
VISIBILITY	Visibility that a policy could foster
MANAG_SUST	Sustainable management of the policy implementation process and its results
COSTS	Necessary costs to activate a policy
COVID19/TOU	Sustainable tourism support according to the current post-COVID19 measures
TOUR_ATTRA	Capacity to attract both tourists and visitors
IV_CONSIST	Consistent integration of the site components and between the site and the architectonic, historical and morphological values of the context, as an Intangible Value (IV) of Olivetti’s vision
IV_SOC_INT	A fruition that would facilitate social integration, with spaces and activities that would generate opportunities for underprivileged families, as an interpretation of the Community Idea of integration between spaces and daily life activities (IV)
IV_DES_CUL	Integration of spaces of a different nature as a possible interpretation of the intangible “design culture” value, which mediates between beauty and functionality

The policies are sometimes clearly grouped (e.g. in two parts, as in MANAG_SUST or in COST, or four, as in IV_CONSIST or in VISIBILITY), but are often not clearly distinguished, such as in IV_DES_CUL. The *Maximising the events* (max_events) policy is always the worst, or one of the worst, while CA TIMESPAN indicates this policy as the best. On the other hand, the *Mainly working on the communication of Olivetti’s legacy values* (communic) policy is always the best or the second best, while CA INVESTMENTS indicates this policy as the worst.

A policy that combines *Maximising the events* of the site and *Mainly working on the communication of Olivetti’s legacy values* (maxev + com) is interesting in terms of VISIBILITY and TOURIST ATTRACTION, but this combined message may generate confusion for the end-users. Anybody who receives communication on events pertaining to music, dance or sport, should combine this communication with that on the intangible values of Olivetti’s legacy. The *Re-activation of the whole site* is combined more easily with *Mainly working on the communication of the legacy values* than with *Maximising the events.* The combination of *Re-activation of the whole site* and *Mainly working on the communication of the legacy values* is almost always the best option, but is only in the second position for Visibility, Costs and Touristic attraction, and not so interesting in terms of Timespan.

These CAs underlined that a combination of different policies and specific enhancement plans could generate interesting results. A combination of the three individual policies should be able to include projects of a different nature and satisfy the different actors’ expectations. However, the associated projects should be coordinated through a systematic and overall vision of the situation of the site, and communication should be activated early on and with a great deal of care.

### From organised knowledge to MC modelling

The compatibility analyses in Fig. 5 suggested a need to further explore the possible cultural and leisure activities that could be included in the UNESCO site, by distinguishing them in at least three groups (activities that can attract a multitude of people for *music, dance or sport events*, or a large number of people, for *exhibitions, theatre, films or* to have *access to documents*, or a handful of people *for sports and recreation*) and by analysing their compatibility with a set of buildings and open-air spaces in the UNESCO site. Fifty-three combinations resulted to be compatible, some spaces resulted to be flexible and could be associated with functions of a different nature, while others could only be associated with one or few functions. The resulting fifty-three compatible combinations were used as input for an MC model and a method application, which verified the potential of using the available knowledge to assign possible actions (combinations of spaces and activities) to ordered risk categories, in relation to aspects included in the CAs (Curto et al., 2022b).

Furthermore, some conclusions of the public policy analysis suggested another analysis, in relation to some possible enhancement projects. Two projects were recent proposals, while three were partially studied during the laboratory activities (Coscia & Curto, 2017; Curto et al., 2018). The projects, and the associated cultural or leisure activities, were logically associated with open spaces that had resulted to be compatible with the activities in the pilot study. A sixth project was also included in the set as a combination of a recent proposal and any of the buildings that had resulted to be compatible with this proposal in the pilot study. The projects were considered possible enhancement actions that needed to be evaluated by an MC model, while an MC method was used to compare and rank the actions, from the most to the least urgent action for the enhancement of the whole site. An MC model was therefore created, and the six projects were evaluated in terms of organisational and economic feasibility, and in relation to the aims of attracting tourism and transferring Olivetti’s values, concepts that had already been analysed as comparing areas.

The model, which is presented in the next section, was created from a technical point of view and was presented to the Municipality, the main actor of the enhancement process, as an example of how projects, plans or strategies could be evaluated.

## Multicriteria modelling and applications of an ELECTRE method

An MC model includes a set of possible actions and a family of coherent criteria. Parameters are introduced into a model to express the preference system of the decision maker(s) (Roy, 1996). If a model is generated to describe how the available knowledge is used to evaluate actions and to test the quality, completeness and usability of the knowledge, such parameters have to be introduced from a technical point of view, in a simulative way that has to be documented and logically explained.

The logical structure of an evaluation model includes (a few) main aspects (or model dimensions) and their analytical formalisation in criteria pertaining to the different related dimensions (Norese, 2016). The structure and size of a model should be simple and minimal to facilitate the model presentation and discussion, although the model considers all the issues, which in this case are outlined in Fig. 4.

**Fig. 7:**
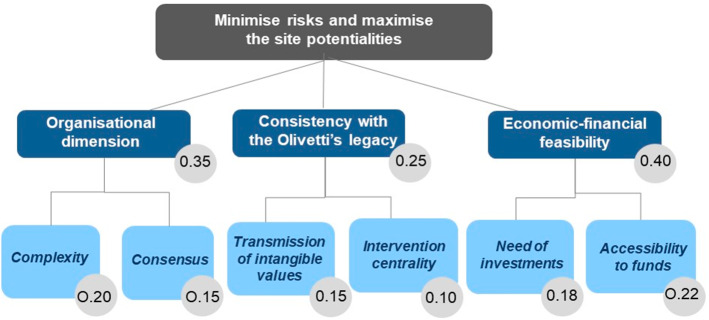
Structure of the model (Source: elaborated by the authors)

An MC model with three dimensions and six criteria (Fig. 7) was used to describe how the six possible projects could be evaluated in relation to criteria that could be associated with different scales and qualitative or quantitative evaluations (Figueira et al., 2005; Roy, 1990).

The first dimension is associated with certain *organisational* risks*,* which cannot be too high, and are dealt with in the MC model by means of two criteria. The second dimension is the *Consistency with Olivetti’s legacy* and includes two criteria, one for the intangible values and the other in relation to a conclusion from the Compatibility and the Comparing analyses: the enhancement of the site implies its whole re-activation and an overall view and communication of the heritage system. Therefore, the centrality of the first projects may be essential. The third dimension is *Economic-financial feasibility*, which has to be considered to evaluate the required investments and the potential attraction and funding from private and public players (two criteria).

The proposed model structure facilitated the description of an essential concept, the relative importance of the criteria, which involves translating a system of values into a set of criteria and in defining their weights.

The values assigned to such parameters have a subjective nature and can only be grasped through communicating with the decision maker(s) in a DA process (Roy & Mousseau, 1996). When the description of a technical point of view is proposed in a DIKEDOC application, these parameters can only be introduced as an example, and are only oriented to balance the structure of the model. The model dimensions represent strategic aspects of the problem. Therefore, a single strategic aspect cannot be much more important than the others or much less important. They may have the same importance or even have a different (but not so different) level of importance, which indicates a possible scenario or a technical point of view. The relative importance of the criteria can be considered a distribution of the importance of a strategic aspect over the associated criteria. This technical approach to the definition of the relative importance of the criteria can be proposed to stakeholders participating in a discussion and used to describe other approaches for decision contexts, and scenario analysis logics.

The six criteria were associated with scales, whose meanings are described hereafter, together with the criteria. In four of the six criteria, the evaluation states of the ordinal scales result from documented combinations of values (see tables in the following sub-sections).

### Organisational dimension

The *Organisational* dimension is dealt with in the MC model by means of two criteria. The first is *Organisational complexity,* which is related to the number and nature of the *Involved actors* and to the *Transformation level* of buildings and open-air spaces that is required to implement any enhancement project. The evaluations of the *Organisational complexity* criterion are the result of a combination of the two factors and their values. The *Involved actors* factor is related to the difficulties that can be generated from a multiplicity of different actors. In relation to the site re-activation, three situations can be distinguished: reduced complexity, when only the Public Administration (PA) is involved; a more complex situation, when some Private actors are involved; and the worst situation, which involves both PA and Private actors. The *Transformation level* factor is related to the nature of the project and can be Low (when the building is not used and the open-air spaces are unimportant, or when all the current uses are confirmed) or Medium (when a part of the building is used or the open-air spaces should be reorganised in order to return to their original use or to improve the effectiveness of the project) or High (when most of the building is occupied and used, and the current uses have to be changed). A logical combination of the involvements of the different actors with the need for changes generates six ordered evaluation states of organisational complexity, which are described in Table 2.

**Table 2:** Ordinal scale of the *Organisational complexity* criterion

Transformation level	Low	Medium	High
Involved actors			
Only PA	1	2	–
Private actors	2	3	5
PA and private actors	3	4	6

The second criterion is the *Consensus* that is easily acquired or maintained if the *Consistency with the citizens’ expectations* and the *Timespan* that is required to clearly manifest the nature of the enhancement project are considered acceptable and the citizens are able to perceive its benefits. The evaluations are the result of the combination of two factors. The *Consistency with the citizens’ expectations* can be High, if services for the citizens are included, or only Medium. A good perception of the nature of the project depends on the quality and rapidity of communication, but a perception that something starts and can produce benefits depends on the complexity of the action and therefore on the *Timespan* necessary to make any action evident. Three time intervals may be considered: less than one year (< 1), between one and two years (1–2) and more than 2 years (> 2). A logical combination of the two factors and their values generates five ordered evaluation states of *Consensus*, which are described in Table 3.

**Table 3:** Ordinal scale of the *Consensus* criterion

Timespan	< 1	1–2	> 2
Consistency with expectations			
High	5	4	3
Medium	3	2	1

### Consistency with Olivetti’s legacy

The potential of any enhancement project to transfer *tangible and intangible values* of the site to the end-users, in order to improve the attractiveness of the UNESCO site, can be dealt with considering two criteria. The built heritage is outstanding, as all the buildings are important for both historical and architectural reasons (tangible values). However, there is a place inside the former factory that has recently been chosen to become the Visitor centre and Info point. The *distance* between any location and this point may have an impact on the enhancement of the site. The evaluations of the *Distance* criterion are not quantitative: the distance from the “heart” of the site could critically reduce the perception of the site as a whole (C—more than 500 m) or may endanger the development of the enhancement of the site (D—100–500 m) or is unessential (U—less than 100 m from the Info point).

The *intangible values* can be *transferred* if the original Community idea of Adriano Olivetti is updated in an overall re-activated site, which allows and supports *Social integration* activities, while *Innovation sustainability* is updated in enhancement projects that involve mediation between beauty and functionality. The evaluations of the *Transmission of intangible values* criterion are the result of the combination of the two factors and their values, which generates four ordered states, as described in Table 4. A complete re-activation of the site that facilitates social integration between the end-users is associated with a Complete Transmission of the intangible value (CT). If the re-activation of the site is only partial, or social integration is not facilitated, there is only a Partial Transmission (PT) and the transmission is Limited if the re-activation of the site is partial and social integration is not facilitated (LT). The *Innovation sustainability* of an intervention is High if the interaction between beauty and functionality is made evident and enhanced through the project, and it is Medium when there is not a clear evidence of this interaction.

**Table 4:** Ordinal scale of the *Transmission of intangible values* criterion

Social integration in an overall re-activated site	CT	PT	LT
Innovation sustainability			
High	4	3	2
Medium	3	2	1

### Economic-financial feasibility

The *Economic-financial feasibility* dimension includes two important criteria, which are aimed at determining the concrete possibility of economically implementing a redevelopment project. The first is the total cost of the *Investment*, which represents the amount of money necessary to restore and redevelop the internal and external spaces of the buildings and reflects the extension of the actions as well as the design complexity related to the newly identified functions. The evaluations are quantitatively based on the estimated amount of money necessary to restore the buildings and their surroundings and to upgrade, integrate or change functions inside and/or outside.

The second criterion is *Potential funding eligibility,* whose evaluations are based on certain assumptions, according to the nature of the enhancement projects. Three of the six actions are related to private projects (a1, a2 and a3), two are related to Public Administration projects (a5 and a6) and the last one is a private project with public participation (a4).

The current economic crisis and the vulnerable socio-economic context in which the site is located (decreasing number of inhabitants, weak real-estate market) do not foster high profitability and increase the investment risk level, thereby reducing the possible role of private enterprises.

In a general scenario of very limited or absent public resources, at least at a local level, the most important source of funding for the public projects implemented at UNESCO sites is the State, and above all Italian Law No. 77/2006 (Special measures for the protection and the exploitation of Italian UNESCO sites).

Therefore, *Potential funding eligibility (PFE)* is an important criterion, but the different nature of the analysed projects makes the elaboration of the criterion difficult.

In the case of private projects, the Internal Rate of Return (IRR) is considered as a proxy of the attraction of real-estate investors and four intervals of IRR values can be associated with four levels of *Attractiveness* (Table 5). In the case of public projects, the availability of state resources and the chance of obtaining funding can instead be hypothesised on the basis of the technical rules (above all 2b on the involvement and participation of the end-users, and 3a on the optimal cost – benefit rate) provided by Italian Law 77/2006, which provides financial support for the enhancement, communication and exploitation of projects. *Chance of funding* is Very high when both evaluations are very high in relation to the 2b and 3a rules. It is only High when one of the two evaluations is very high and the other is sufficient, while it is Medium when both are only sufficient and Low when one of them is low. The PFE criterion scale is associated to four ordered evaluation states, which are described in Table 5.

**Table 5:** Ordinal scale of the *Potential Funding Eligibility* (PFE) criterion

For private projects: attractiveness (IRR)	Very good (≥ 15%)	Good (< 15%, ≥ 11%)	Weak (< 11%, ≥ 7%)	Low (< 7%)
For public projects: chance of funding L.77/2006	Very high	High	Medium	Low
PFE ordinal scale	4	3	2	1

### Evaluation of the projects and results of the ELECTRE II method

The evaluations of the actions, in relation to the six criteria, are included in Table 6, together with the states of the adopted scales and their preference direction, that is, from the worst to the best. Table 6 facilitated a first reading, which underlined how five of the six actions were not efficient because they were dominated by at least one other action (a5). The only efficient, or Pareto optimal, action, a5, could be indicated as the most urgent intervention to start the re-activation of the site, while the relative positions of the other possible interventions were not known, and a ranking of the different actions could be required. An outranking method of the ELECTRE family (Figueira et al., 2005) was chosen to compare and rank the actions.

**Table 6:** Structural elements of the MC model

Criteria [Scale and reference direction]	Organisational complexity g1 [6–1]	Consensus g2 [1–5]	Distance g3 [C,D,U]	Intangible v. transfer g4 [1–4]	Investment g5 Mln € [ −]	Funding eligibility g6 [1–4]
Relative importance (or weights) Projects (A)	0.20	0.15	0.10	0.15	0.18	0.22
a1 OCC_Food & Sport	5	4	D	3	12	1
a2 DPC_Music	3	4	C	1	6	3
a3 SSC_Multifunctional	3	4	U	3	4	2
a4 CHP_Archive	3	1	D	2	8	2
a5 OpenairMuseum	1	5	U	4	2	4
a6 IndoorOpenairMuseum	2	5	U	4	3	3
Discordance sets	[6–1]	[1–5]	–	[1–4]	[12–3, 12–2]	[1–4]

The number of projects that had to be compared was limited. They represented almost homogenous logical proposals, without detailed data, whose evaluations were associated with “good” ordinal scales, and with few clearly distinguished and completely documented evaluation states. Only one cardinal scale, where the net preference was always present between couples of evaluations, was used. Therefore, the model only included true criteria. The application of ELECTRE II (Roy & Bertier, 1973) was considered possible and appropriate in this case.

This was the first ELECTRE method that was designed specifically to deal with ranking problems. It is now only used in rare situations (to rank actions when no uncertainty is associated with the evaluations), but it is an interesting option when the method is only used to describe the role of an MC method in a decision situation, because the application of the method can be described in detail, without the aid of an SW tool, and can be used to explain the logic of any outranking method. Very few parameters have to be defined, and the passage to other ELECTRE methods and their parameters can be postponed until they are required during a DA intervention.

The weights that distinguish the criteria, in terms of relative importance, were the result of a (documented) technical choice of the different levels of importance of the model dimensions (0.35 for Organisational dimension, 0.25 for Consistency with Olivetti’s legacy and 0.40 for Economic-financial feasibility) and the consequent distribution of this importance on the related criteria, as shown in Fig. 7 and Table 6.

The discordance sets were the other non-mandatory parameters. They included couples of values logically in discordance, in relation to criteria where a very bad evaluation of an “interesting” action on a criterion could generate a risky decision whenever another action presented a very good evaluation of the same criterion, In this case, discordance sets were proposed for five criteria but not for the Distance criterion, because the logical distance between the three evaluation states was not so high and a logical discordance did not result to be present.

The ELECTRE II method (see Appendix 1) includes two phases. In the first phase, preferences between couples of actions are modelled by means of the binary outranking relation S which, in this ELECTRE method, means the application of two tests, that is, the concordance test and the non-discordance test. Outranking relation S can be represented by an outranking graph and the second phase activates two iterative procedures on the graph, a descending procedure, which is oriented towards identifying, at each iteration, "the actions that are not outranked”, and an ascending procedure, which is oriented towards identifying “the actions that do not outrank any other action”.

The ELECTRE II application (see Appendix 2) produced the same sequence of classes, in the ascending and descending procedures, from the most urgent action to the least urgent, that is {a5}, {a6}, {a3}, {a2}, {a4} and {a1}. The only efficient, or Pareto optimal action, a5, resulted to be the priority action, while the order of the other projects suggested a possible activation sequence, which could be obtained by means of the implementation of an integrated set of different but coherent policies.

## The pilot study results

The study was conducted on behalf of the Municipality of Ivrea, even though a large number of public and private stakeholders were involved in the past and could also be involved in a future decision process. The aim of the interdisciplinary team was to describe how knowledge about the site enhancement problem could be synthesised from the main public actor’s point of view, but also to underline the need to consider the different goals and expectations of the other actors, at least as criteria for new models.

What had been a confused list of questions became a structured identification of issues, which in turn facilitated the formulation of the problem that the strategic planning phase of the enhancement process could or should study and deal with.

The Compatibility analyses were applied in relation to some different decision problems, reduced the complexity of the problem and facilitated the assignment of the several compatible actions to risk categories and the elaboration and comparison of some possible actions, i.e. enhancement projects aimed at restoring and redeveloping buildings and open-air spaces by installing suitable new functions that would be able to attract public and private investments and re-activate the whole site.

The conceptual use of the Comparing Areas fostered a detailed analysis of some of the complicated concepts, mainly the intangible values, and the translation of concepts into formal expressions of values, that is, criteria. Furthermore, it also facilitated the definition of the main policies the Municipality could adopt to enhance the UNESCO site.

In a DIKEDOC application, MC models transparently synthesise the knowledge that has been acquired, integrated and organised, allow possible decisions to be analysed, and elements of knowledge to be redefined and improved.

The formal modelling of some criteria underlined the need for new knowledge, which had not been generated during the laboratory activities, and was inferred from press reviews or from events and seminars that took place during the pilot study. This knowledge was debated, linked to the previously integrated knowledge and used by the interdisciplinary team. The evaluation of the projects was supported through the use of a dynamic GIS, developed during the laboratory activities and updated in the pilot study, and which could be used to assist the Municipality of Ivrea and the stakeholders in developing compatible strategies, policies and actions for the enhancement of the whole site.

MC outranking methods were used to describe how the available knowledge can be used to deal with some problems in a decision context, which results can be produced, and which elements of a preference system should be constructed in the decision aid process (Norese, 2009). The ELECTRE applications produced results that were considered examples of possible decision activities (to exclude risky actions or clarify the possible roles of some specific decisions in the enhancement process).

The interdisciplinary team considered the results of the pilot study, at the technical level, as an interesting proposal and a starting point to foster the activation of the second phase, at a participative level, to test the DIKEDOC methodology.

The procedural logic of the study and some results were to have been presented in a round-table meeting with some potential actors in the UNESCO site enhancement process. The meeting was organised and planned for April 2020, but it was not conducted because the COVID-19 emergency created urgent new problems and interrupted the organisation of any event involving direct participation. New contacts were activated in Autumn, and in January 2021 some procedural steps and preliminary results were presented during a meeting with the Mayor of the Municipality of Ivrea and the Coordinator of the UNESCO site, who had also been the Coordinator of the UNESCO Nomination process.

The pilot study was proposed as a logical and analytical “description” of the Municipality’s space of action, in relation to different policies and enhancement projects. Some tools, together with their applications in the pilot study, and some preliminary results were illustrated to point out the potentiality of the methodology, in relation to the complexity of the enhancement process and to the crisis that the COVID-19 emergency has generated in the tourism sector, especially for the role that any Municipality could play in relation to the measures to implement the Next Generation EU program in Italy. The interdisciplinary team proposed two workshops, the first with the Coordinator of the UNESCO site, some potential actors from the territory and the owners of the private assets, in order to present the methodology and discuss its preliminary results; the second with the Mayor of Ivrea and the council members, plus other public organisations included by the Municipality, in order to test the reaction of the parties, in relation to the technical proposal.

The logic of the methodology resulted clear and the results convincing. The participants appreciated both the models and the methods. The Coordinator of the UNESCO site proposed using MC models and methods to ex post validate decisions, to facilitate the monitoring requested by UNESCO and to contribute to the Heritage Impact assessment (HIA) procedure and the definition of the Strategic conservation plan (SCP). The Municipality proposed the HIA and the SCP documents for the analysis of the interdisciplinary team and the team asked to be put in contact with the team involved in the HIA and SCP procedures.

The documents arrived after some weeks, but they only included some ideas about the definition of bureaucratic procedures. A subsequent series of contacts with the Municipality clarified the situation. The proposal of two workshops was not taken into consideration and any relationship between the analysts and the team involved in the HIA and SCP or any other organisations of the territory was discouraged. It was also underlined that the role of the Public Administration in the enhancement of the UNESCO site had been and would remain minimal or even absent, in relation to the public spaces, while private organisations were and would be active in proposing and activating projects.

Any contact between the Politecnico di Torino and the Administration was interrupted in March 2021, one year before the next administrative election, when the Municipality will have to answer the question “How was the enhancement of the UNESCO site planned and implemented?”.

## Conclusions

DIKEDOC is a methodology that integrates logical and analytical tools to be used first at a technical level, with knowledge sources of any nature, and then in a participative context in relation to different situations (from a committee to a table of experts, from a workshop to a round-table meeting or a virtual space, and so on). Its versatility was verified in the pilot study and, above all, during several preparatory MCDA interventions in innovative situations where the activation of a decision process was considered a risk, with experts or stakeholders, and in technical and political committees, with scientific and/or technical knowledge sources and potential decision makers.

DIKEDOC can be proposed at a technical level to provide a shared context for people, who may be experts, skilled practitioners and/or more in general, sources of knowledge, to organise dispersed knowledge in a way that generates understanding and insights, and which can be communicated, at a participative level, to enable dialogue with people who are stakeholders in a decision problem where a decision system is latent or not yet activated. At a participative level, the aim of DIKEDOC is to create an opportunity to interact, share personal points of view and experiences, explore spaces of action, where the tools facilitate understanding, criticism and inclusion of proposals and changes, and to create new knowledge. DIKEDOC stimulates learning at both of these levels, i.e. the construction of understanding and the emerging of a community of interpretation (Brown & Duguid, 1991), and collaborative processes based on a constructive vision of decision aiding. However, DIKEDOC is more effective than efficient, because significant results can be acquired but, in general, time and effort necessary to conduct such methodology are not minimal.

The lessons learned and the results achieved from the pilot study have underlined that DIKEDOC can be proposed to an interdisciplinary team of knowledge sources who agree to interact and share their expertise, experience or understanding of data, facts and histories. The pilot study was organised to communicate knowledge and ways of using such knowledge in the complex social context within which the UNESCO site has been perceived as an opportunity, if its enhancement takes place.

The application of DIKEDOC at a participative level can be proposed to stakeholders who are willing to participate in discussion groups or workshops that should only be considered as preparatory events, where conflictual visions may be expressed, either directly or indirectly, without the activation of the “fighting arena” factor. When DIKEDOC is applied in formal committees, its main aim is that of reducing the conflictual atmosphere and frequent misunderstandings, and of facilitating the passage towards a collaborative attitude (see Norese & Toso, 2004; Norese, 2006).

Logical and analytical tools can be used as procedural references that reduce ambiguities and inappropriate behaviour, and the software tools used to apply decision aid methods allow the participants to visualise problems and model structures and to understand procedures and results. However, the participation in and generation of an interpretation community, which discusses and proposes legitimate expressions of knowledge and possible actions, must be legitimated. This legitimation was initially agreed upon in the pilot study but then made impossible.

The DIKEDOC methodology is currently being applied in relation to a new case, that is, the reuse of a large area, built in 1935, in the centre of Turin and which has till recently been used as a hospital. Other possible applications could be implemented in relation to the activation of the National Recovery and Resilience Plan—called “Recovery Plan”. The Recovery Plan represents a great opportunity for Public Administrations, which have to adequately identify, select and estimate potential enhancement/redevelopment projects, as well as to properly describe and communicate them in order to be funded. Therefore, DIKEDOC could support Public Administrations in these difficult tasks. However, legitimation should always be discussed at the start of any intervention and constructed during its activation.

## Figures and Tables

**Fig. 8:**
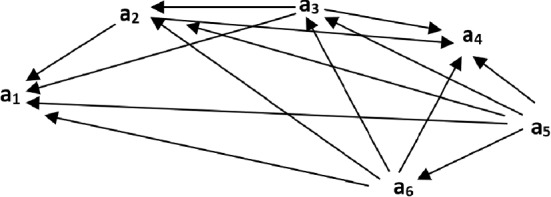
Outranking graph ( Source: elaborated by the authors)

## Data Availability

The data used in the model are transparent; other data and materials are not available. Not applicable.

## Appendix 1

### ELECTRE II

The ELECTRE II method is an outranking method that can be used to deal with the problem of ranking a set of actions from the best option to the worst (Figueira et al., 2005) in the classification problem statement. Like the other ELECTRE methods, ELECTRE II includes two phases: construction of an outranking relation, S, whose meaning is *at least as good as*, followed by a procedure that applies a decision rule that is consistent with the specific decision problem and is used to elaborate recommendations from the results obtained in the first phase. The ELECTRE II method is applied to an MC model whose components are: A, a complete set of actions a_i_ ∈ A; a family J of consistent criteria g_j_ ∈ J, which associates, to each a_i_ ∈ A, its evaluation, g_j_(a_i_) ∈ E, in relation to a specific criterion g_j_ and its scale E, and inter-criterion parameters.

### First phase of ELECTRE II

The outranking relation S is a binary relation that is used to model preferences between couples of actions. Considering two actions, a and a’, four situations may occur: aSa’ and not a’Sa, i.e., aPa’ (a is strictly preferred to a’); a’Sa and not aSa’, i.e., a’Pa (a’ is strictly preferred to a); aSa’ and a’Sa, i.e., aIa’ (a is indifferent to a’); not aSa’ and not a’Sa, i.e., aRa’ (a is incomparable to a’). If one of the P or I situations is verified, there is outranking. If neither P nor I are verified, there is incomparabily, R, a preference relation that is useful to account for situations in which the decision maker is not able to compare two actions. The ELECTRE II method can only be applied if each criterion is a true-criterion, for which there is strict, or net, preference for each difference between evaluations and indifference when the evaluations are the same. The outranking relation is based on the concordance–discordance principle, which involves declaring that an action is at least as good as another if a “majority” of the criteria supports this assertion (concordance condition) and if the opposition of the other criteria does not generate “too strong” reasons (non-discordance condition). An outranking relation is constructed with the aim of comparing, in a comprehensive way, each pair of actions (a,a’), and the concordance–discordance principle is implemented in ELECTRE II by means of two tests that verify concordance and non-discordance conditions.

#### Concordance test

An action a can outrank an action a’, aSa’, if a sufficient majority of criteria are in favour of this assertion. The concordance condition can be defined as follows: the concordance index C(aSa’) has to be at least equal to a concordance level c, and C(aSa’) has to be at least equal to C(a’Sa), in order to consider only conditions of preference and not of indifference. In order to make this definition operational, the criteria are partitioned into J + , which includes the criteria in favour of the first element of the couple (a, a’), J = (when the evaluations of a and a’ are equal) and J-, the criteria in favour of the second element of the couple (a, a’). The weights pj of the criteria included in J^+^, J^=^ and J^−^ are synthesized in P^+^, P^=^ and P^−^.

$$ \begin{aligned} {\text{P}} ^{+} \left( {{\text{a}},{\text{a}}^{\prime}} \right) 
= \sum\nolimits_{{\text{j}} \in {\text{J}^{+}}}  {\text{pj}} \\ {\text{P}} ^{=} \left( {{\text{a}},{\text{a}}^{\prime}} \right) = \sum\nolimits_{{\text{j}} \in {\text{J}^{=}}}  {\text{pj}} \\ {\text{P}} ^{-} \left( {{\text{a}},{\text{a}}^{\prime}} \right)  = \sum\nolimits_{{\text{j}} \in {\text{J}^{-}}}\, {\text{pj}} \\ \end{aligned} $$

These weights are used in the concordance test:

$$ \begin{aligned} {\text{C}}\left( {{\text{a}},{\text{a}^{\prime}}} \right) & = {\text{P}} + \, \left( {{\text{a}},{\text{a}^{\prime}}} \right) \, + {\text{ P}} = \, \left( {{\text{a}},{\text{a}^{\prime}}} \right) \, / \, \Sigma {\text{Pj }} \ge {\text{c }}\left( {\text{level of concordance}} \right) \\ {\text{P}} + \, \left( {{\text{a}},{\text{a}^{\prime}}} \right) & \ge {\text{P}}^{ - } \left( {{\text{a}},{\text{a}^{\prime}}} \right) \\ \end{aligned} $$

#### Non discordance (or veto) test

When the concordance condition holds, none of the criteria in the minority should oppose the assertion aSa’ too much. In order to make this definition operational, a set of discordance Dj* is created to include couples of values (e, e’) that are considered too discordant (e is “too much” worse than e’) in relation to the J* criteria, which can activate the discordance test (the test can be activated in relation to all the criteria, but also in relation to just some of them). If (a, a’) is a couple of actions and their evaluations are

$$ {\text{g}}_{{{\text{j}}*}} \left( {\text{a}} \right) = {\text{e}}\,{\text{and}}\,{\text{g}}_{{{\text{j}}*}} \left( {\text{a}^{\prime}} \right) = {\text{e}^{\prime}} $$

for at least one of the J* criteria, a does not outrank a’, even though the concordance test for the couple (a, a’) has been passed.

### Second phase of ELECTRE II

The outranking relation S, which is constructed in the first phase, can be represented by an outranking graph, where the actions are the nodes and the oriented arcs indicate the presence of an outranking relation between two nodes. The second phase activates two iterative procedures on the graph to produce two preorders (i.e. orders that accept an element in joint position with others in some classes). The first (descending) procedure is oriented toward identifying, at each iteration, a sub-set of actions (D_i_) that follow this rule: "the best actions are not outranked”. The second (ascending) procedure is oriented toward identifying actions that follow this different rule: “the worst actions do not outrank any other action”.

If the graph does not include circuits, at least one action is consistent with the procedure rule at each iteration. When only one action is consistent with the rule, it is assigned to a class (C^±^) and eliminated from the graph. When more than one action is identified by the rule, a weak outranking relation is applied, by means of a weak concordance level, c^w^, to the sub graph that only includes the actions of D_i_. The same rule is then applied to the sub graph.

The two procedures produce the same result or results that are marginally or substantially different. At the end of the second phase, the intersection of two marginally different preorders produces a final partial graph (some remarks on the analysis of these graphs and on the cases of different results have been proposed in Norese et al., 2016).

## Appendix 2

### The ELECTRE II application

The ELECTRE II method (see the Appendix 1) included two phases, and in the first phase, preferences between couples of actions were modelled by means of the binary outranking relation S that, in this ELECTRE method, means the application of two tests, the concordance and the non-discordance tests. The application of the first phase of ELECTRE II produced the results synthesised in Table

**Table 7:** First phase with the concordance and non discordance tests

(a,a’)	J^+^	J^=^	J^–^	P^+^ ≥ P^–^	P^+^ + P^=^	ND test	S
a_1_ a_2_	3,4	2	1, 5, 6	No			
a_1_ a_3_	/	2, 4	1, 3, 5, 6	No			
a_1_ a_4_	2, 4	3	1, 5, 6	No			
a_1_ a_5_	/	/	1, 2, 3, 4, 5,6	No		Yes	
a_1_ a_6_	/	/	1, 2, 3, 4, 5, 6	No		Yes	
a_2_ a_1_	1, 5, 6	2	3, 4	Yes	0,75		S
a_2_ a_3_	6	1,2	3, 4, 5	No			
a_2_ a_4_	2, 5, 6	1	3, 4	Yes	0,75		S
a_2_ a_5_	/	/	1, 2, 3, 4, 5, 6	No		Yes	
a_2_ a_6_	/	/	1, 2, 3, 4, 5, 6	No		Yes	
a_3_ a_1_	1,3,5,6	2, 4	/	Yes	1		S
a_3_ a_2_	3, 4, 5	1,2	6	Yes	0,78		S
a_3_ a_4_	2, 3, 4, 5	1, 6	/	Yes	1		S
a_3_ a_5_	/	3	1, 2, 4, 5, 6	No			
a_3_ a_6_	/	3	1, 2, 4, 5, 6	No			
a_4_ a_1_	5, 6	1, 3	2, 4	Yes	0,70		
a_4_ a_2_	3, 4	1	2, 5, 6	No			
a_4_ a_3_	/	1, 6	2, 3, 4, 5	No			
a_4_ a_5_	/	/	1, 2, 3, 4, 5, 6	No		Yes	
a_4_ a_6_	/	/	1, 2, 3, 4, 5, 6	No		Yes	
a_5_ a_1_	1, 2, 3, 4, 5,6	/	/	Yes	1		S
a_5_ a_2_	1, 2, 3, 4, 5, 6	/	/	Yes	1		S
a_5_ a_3_	1, 2, 4, 5, 6	3	/	Yes	1		S
a_5_ a_4_	1, 2, 3, 4, 5, 6	/	/	Yes	1		S
a_5_ a_6_	1, 5, 6	2, 3, 4	/	Yes	1		S
a_6_ a_1_	1, 2, 3, 4, 5,6	/	/	Yes	1		S
a_6_ a_2_	1, 2, 3, 4, 5, 6	/	/	Yes	1		S
a_6_ a_3_	1, 2, 4, 5, 6	3	/	Yes	1		S
a_6_ a_4_	1, 2, 3, 4, 5, 6	/	/	Yes	1		S
a_6_ a_5_	/	2, 3, 4	1, 5, 6	No			

7: partition of the criteria family into J^+^, J^=^ and J^−^, levels of concordance P^+^  + P^=^, when P^+^ is at least equal to P^–^, and the presence of discordance as a result of the Non-Discordance (ND) test. They were then transferred in the outranking graph in Fig. 8, where each arc represented one of the outranking relations modelled in the first phase, in relation to the strong concordance level c^S^ = 0.75.

In the second phase, the descending (P(A)^+^) and ascending (P(A)^−^) procedures were applied to the outranking graph of Fig. 8, which does not include circuits.

#### The descending procedure to create a classification from the best to the worst (P(A) +)

The actions that are not outranked (D_i_) are identified and analysed in order to be assigned to class Ci^+^ and eliminated from the graph at each iteration i.

- Iteration 1: A1 = A
- D_1_ = {a5} and C^1+^  = {a5}
- Iteration 2: A^2^ = A^1^\C^1+^  = {a1, a2, a3, a4, a6}
- D_2_ = {a6} C^2+^  = {a6}
- Iteration 3: A^3^ = A^2^\C^2+^ = {a1, a2, a3, a4}
- D_3_ = {a3} C^3+^  = {a3}
- Iteration 4: A^4^ = A^3^\C^3+^ = { a1, a2, a4}
- D_14_ = {a2} C^4+^  = {a2}
- Iteration 5: A^5^ = A^4^\C^4+^ = {a1, a4}
- D_5_ = {a1, a4}
- D_5_ includes the two actions that are not outranked. The weak outranking relation is activated in order to distinguish between the actions. It adopts the weak concordance level c^W^ = 2/3 in the concordance test, in relation to the sub-graph, which only includes the actions of D5. The weak outranking relation can distinguish between the actions: a4 is the only action that is not outranked, and only this action is therefore assigned to class C^5+^ of the descending pre-order.

- C^5+^  = {a4}
- Iteration 6: A^6^ = A^5^\C^5+^ = {a1}
- C^6+^  = {a1}
- Iteration 7: A^7^ = A^6^\C^6+^ = Ǿ →|A^7^|= 0 STOP
- P(A)^+^ (sequence of the classes from the best to the worst) = {a5},{a6},{a3},{a2},{a4},{a1}

#### The ascending procedure to create a classification from the worst to the best (P(A)^–^)

The actions that cannot outrank any other action (D_i_) are identified and analysed in order to be assigned to class C^i−^ and eliminated from the graph at each iteration i.

- Iteration 1: A1 = A D_1_ = {a1, a4}
- C^1–−^ = {a1}
- Iteration 2: A^2^ = A^1^\C^1−^ = {a2, a3, a4, a5, a6}
- D_2_ = {a4} and C^2−^ = {a4}
- Iteration 3: A^3^ = A^2^\C^2−^ = {a2, a3, a5, a6}
- D_3_ = {a2} and C^3−^ = {a2}
- Iteration 4: A^4^ = A^3^\C^3−^ = {a3, a5, a6}
- D_4_ = {a3} and C^4−^ = {a3}
- Iteration 5: A^5^ = A^4^\C^4−^ = {a5, a6}
- D_5_ = {a6} and C^5−^ = {a6}
- Iteration 6: A^6^ = A^5^\C^5−^ = { a5}
- D_6_ = {a5} and C^6−^ = {a5}
- Iteration 7: A^7^ = A^6^\C^6−^ = Ǿ →|A^8^|= 0 STOP
- P(A)^−^ (sequence of the classes from the worst to the best) = {a1},{a4},{a2},{a3},{a6},{a5}

##### Results

The ascending and descending procedures produced the same sequence of classes, from the most urgent action to the least urgent, that is {a5 OpenairMuseum}, {a6 IndoorOpenairMuseum}, {a3 SSC_Multifunctional}, {a2 DPC_Music}, {a4 CHP_Archive} and {a1 OCC_Food&Sport}.
